# Evidence for temporal disintegration of information processing during sensorimotor integration in GTS

**DOI:** 10.1016/j.nicl.2025.103844

**Published:** 2025-07-13

**Authors:** Yifan Hao, Paul Wendiggensen, Annet Bluschke, Tina Rawish, Julia Friedrich, Eszter Tóth-Fáber, Zsanett Tárnok, Veit Roessner, Christian Frings, Anne Weissbach, Tobias Bäumer, Alexander Münchau, Christian Beste

**Affiliations:** a Institute of Systems Motor Science, University of Lübeck, Lübeck, Germany; b Cognitive Neurophysiology, Department of Child and Adolescent Psychiatry, Faculty of Medicine, TU Dresden, Dresden, Germany; c Brain, Memory and Language Research Group, Institute of Cognitive Neuroscience and Psychology, HUN-REN Research Centre for Natural Sciences, Budapest, Hungary; d Institute of Psychology, ELTE Eötvös Loránd University, Budapest, Hungary; e Vadaskert Child and Adolescent Psychiatry Hospital and Outpatient Clinic, Budapest, Hungary; f German Center for Child and Adolescent Health (DZKJ), Partner Site Leipzig/Dresden, Dresden, Germany; g Cognitive Psychology, University of Trier, Trier, Germany

**Keywords:** Tourette Syndrome, EEG, Sensorimotor integration, Event file, Theta, Alpha, Beta

## Abstract

In Gilles de la Tourette syndrome (GTS), a neuropsychiatric disorder defined by the presence of tics, bindings of perceptual and motor processes in what cognitive theories refer to as event files is altered. The neural basis of such abnormal coupling though is currently unclear, particularly as regards oscillatory activity in theta, alpha, and beta frequency bands. The inter-relation between oscillatory activities in the theta, but also the alpha and beta band during event file binding and retrieval was investigated in the present study in patients with GTS and healthy controls (HC) using a well-established stimulus–response event file task and concomitant EEG recording. Behaviorally, binding effects did not differ between groups. Also, there were well-known patterns of theta, alpha and beta band activity during retrieval in both groups. In addition, corroborating previous findings in HC, in the period after event file binding and before retrieval, theta, alpha and beta band activity was found in the insula cortex, inferior/middle frontal and superior/middle temporal areas of the right hemisphere in both groups. However, in comparison to HC, GTS patients exhibited relatively less widespread oscillatory correlations between the post-binding and retrieval periods. Notably, a correlation between beta-band oscillations in the supplementary motor area (SMA) and oscillatory activity during the post-binding period was observed only in HC, but not in GTS. This absence in GTS may reflect a disrupted management of event files following retrieval. Overall, the findings suggest a relative decoupling of oscillatory activity associated with binding and retrieval processes in individuals with GTS.

## Highlights

• Perception-action integration (PAI) in Tourette Syndrome (GTS) is examined.
• The relevance of theta, alpha and beta band activity for PAI in GTS is uncovered.
• GTS patients reveal a temporal disconnect of activity during PAI.

## 1 Introduction

Gilles de la Tourette Syndrome (GTS) is a common neuropsychiatric disorder primarily defined by the presence of tics (Johnson et al., 2023, Robertson et al., 2017). Although traditionally categorized as a movement disorder (Beste and Münchau, 2018), this view has increasingly been challenged (Bartha et al., 2023, Paulus et al., 2021). One emerging perspective conceptualizes GTS as a disorder marked by excessively strong bindings between sensory and motor processes (Beste and Münchau, 2018), which cognitive theories refer to as so-called event files (Hommel, 2004). Evidence supporting the view that GTS can be conceptualized as a disorder of altered event file dynamics (i.e. integrated sensorimotor representations) has accumulated in a number of studies suggesting that coupling of perceptual and motor processes may be abnormally increased in GTS (Adelhöfer et al., 2021, Kleimaker et al., 2020b, Petruo et al., 2019, Petruo et al., 2020), the neurophysiological underpinnings of which are far from being understood. This uncertainty is particularly relevant when considering neural oscillatory activity involved in brain information processing (Fries, 2015) and recent theories regarding the roles of theta, alpha, and beta frequency bands in perception–action integration within fronto-parietal cortices (Beste et al., 2023) that are of known relevance to the pathophysiology of GTS (Church et al., 2009, Naro et al., 2020).

Concepts on the role of theta, alpha and beta oscillations (Beste et al., 2023) closely connect to the so-called “binding and retrieval in action control (BRAC)” framework (Frings et al., 2020, Frings et al., 2024). In brief, the BRAC framework specifies the operation principles and the dynamics, with which integrated representations of sensorimotor codes, i.e. event files, shown to be important for the understanding of GTS, are handled. An important aspect of BRAC is that the processing of event files depends on the immediate past or the state of the neurophysiological system, which is typically investigated in prime-probe structured experiments (Frings et al., 2020). In the latter, a sensorimotor representation (i.e. an event file) is build/bound at the time point of a prime (S1) and consecutively retrieved at the time of the probe stimulus (S2). The explicit distinction between binding (i.e., integration of stimulus and action features occurring in temporal proximity into an event file) and retrieval of event files (when an event file is reactivated through re-encountering at least one of the elements included in the event file), has been corroborated by many studies (Frings et al., 2024). These studies also suggest that there is close reliance of processes occurring at the retrieval stage on processes that occurred at the time of initial event file creation/binding (Frings et al., 2024, Schmalbrock et al., 2023).

Notably, it has been suggested that for binding and retrieval processes particularly theta band activity is of importance (Beste et al., 2023). Recent empirical evidence corroborates this (Mayer et al., 2025, Rawish et al., 2024). The relevance of theta band activity during these processes is in line with its role during action control (Cavanagh and Frank, 2014) and memory-related retrieval processes (Roux and Uhlhaas, 2014). Theta band activity has long been discussed to play an essential role in the pathophysiology of GTS, especially with respect to the generation of tics (Alam et al., 2015, Cagle et al., 2020, Giorni et al., 2017, Jimenez-Shahed et al., 2016, Maling et al., 2012, Marceglia et al., 2010, Molina et al., 2018, Münchau et al., 2021, Neumann et al., 2018, Priori et al., 2013, Shute et al., 2016). Given the role of theta band activity during binding and retrieval processes of event files (Beste et al., 2023) and the usefulness of event file dynamics as a framework for GTS (Kleimaker et al., 2020a), it is of interest to more closely examine the modulation of theta band activity in GTS during binding and retrieval processes.

Evidence suggests that there is a close interrelation (correlation) between theta band activity pattern in functional neuroanatomical regions active at the time of creation/binding of an event file and the time point of its retrieval (Rawish et al., 2024). It has been shown that following binding of an event file until its retrieval regions in the insular cortex, anterior temporal lobe and inferior frontal gyrus maintain event file-related processes and that activity in these regions is systematically related to activity in (other) cortical regions involved in the retrieval of the event file (Mayer et al., 2025, Rawish et al., 2024). The question of how binding and retrieval processes during sensorimotor integration are modulated in GTS therefore touches aspects of connectivity patterns and their modulations in these patients.

Several lines of evidence suggest that GTS is associated with a relative disconnect between regions engaged in sensorimotor integration (Church et al., 2009, Naro et al., 2020), but the pattern of findings is inconclusive. Both decreased and increased functional connectivity patterns have been observed depending on the brain regions and age of individuals included in the data analysis (Jurgiel et al., 2021, Nielsen et al., 2020, Polyanska et al., 2017, Ramkiran et al., 2019, Worbe et al., 2012). It is, therefore, equally probable that there is higher or lower inter-relation between theta band activities during event file binding and retrieval in GTS.

According to recent concepts of how event files are dynamically handled, theta band activity holds indeed a central position (Beste et al., 2023). However, it should not be viewed independently from alpha and beta band activity. Indeed, there is evidence that alpha and beta band activity are evident during the maintenance of event files (i.e. between binding and retrieval processes) (Rawish et al., 2024). In particular, alpha band activity has recently been shown to be closely linked to theta band activity during dynamic event file management (Pastötter and Frings, 2023, Wendiggensen et al., 2023). Therefore, the present study investigates how theta band activity is modulated during the dynamic management of event files in GTS but also considers alpha and beta band activity in these processes.

## 2 Material and methods

### 2.1 Participants

The analysis included N = 116 participants, consisting of 54 individuals (35 males, 19 females) who had been clinically diagnosed with GTS and 62 HC (37 males, 25 females). The age range for the GTS group was 8 to 53 years (*M* = 21.57, *SD* = 11.38), and for the HC group 8 to 49 years (*M* = 20.08, *SD* = 10.17). Detailed demographic comparisons between the GTS and HC groups are presented in Table 1. There were no significant differences between the two groups in terms of sex, handedness, and age, while a significant difference in IQ was observed between the two groups (GTS: 105.27 ± 12.38, HC: 110.21 ± 10.73, *t*(100) = −2.22, *p* = 0.03).

**Table 1:** Demographic and clinical characteristics of participants.

Participant characteristic	GTS (n = 54)	HC (n = 62)
**Demographics**		
Sex (male/female)	35/19	37/25
	*χ^2^* = 0.14, *p* = 0.71	
Handedness (right/left)	46/7	56/6
	*χ^2^* = 0.09, *p* = 0.76	
Age	21.57 ± 11.38 (8–53)	20.08 ± 10.17 (8–49)
	*t*(107) = 0.74, *p* = 0.46	
IQ	105.27 ± 12.38 (76–125)	110.21 ± 10.73 (83–134)
	*t*(100) = −2.22, *p* = 0.03	

**Clinical assessment**		
Comorbidity (yes/no)	15/38	2/60
	*χ^2^* = 12.34, *p* < 0.01	
Medication (yes/no)	10/38	1/54
	*χ^2^* = 7.74, *p* < 0.01	
Rush, motor tics/min	37.37 ± 25.03 (3.9–101.6)	–
Rush score (0–20)	10.95 ± 3.60 (3–19)	–
Disease duration (years)	12.47 ± 10.69 (0.25–41)	–
Motor tic frequency score (0–5)	3.35 ± 1.32 (1–5)	–
Vocal tic frequency score (0–5)	2.13 ± 1.84 (0–5)	–
YGTSS, tics (0–50)	18.87 ± 9.72 (4–44)	–
YGTSS, total (0–100)	33.98 ± 18.77 (4–91)	–
DCI (0–100)	52.98 ± 19.56 (24–100)	–
PUTS (0–40)	17.30 ± 10.41 (0–32)	–

*Note:* GTS = Gilles de la Tourette syndrome patients; HC = Healthy control; Rush = Rush Video-Based Tic Rating-Scale; YGTSS = Yale Global Tic Severity Scale; DCI = Diagnostic Confidence Index; PUTS = Premonitory Urge for Tics Scale.

All participants performed the same stimulus (S)-response (R)-binding task, which was conducted in the specialized GTS outpatient clinic of the Institute of Systems Motor Science at the University Hospital Medical Center Schleswig-Holstein, Campus Lübeck, Germany; the Department of Child and Adolescent Psychiatry at the University Hospital Dresden, Germany and the Vadaskert Child and Adolescent Psychiatry Hospital in Budapest, Hungary. The studies were conducted under the same experimental conditions.

Before the behavioral task, participants completed a series of standardized clinical assessments. This comprehensive evaluation included IQ testing, handedness determination, clinical neuropsychiatric interviews, and the assessment of tic severity. IQ was measured using the short German version of the Wechsler Intelligence Scale for Children (HAWIK-IV) (Petermann & Petermann, 2008) for participants aged 9 to 16 and the Wechsler Adult Intelligence Scale (WAIS) (Wechsler, 2012) for those over 16. Handedness was assessed using the Edinburgh Handedness Inventory (Oldfield, 1971). For psychiatric comorbidities screening, including mood disorders, obsessive–compulsive disorder (OCD) and deficit hyperactivity disorder (ADHD), all participants over 17 years of age completed the Mini International Neuropsychiatric Interview (M.I.N.I.), while participants aged 9 to 17 completed the Mini International Neuropsychiatric Interview Kid (M.I.N.I. KID) (Sheehan et al., 1998). Of note, for the GTS patients, lifetime tic presence was evaluated using the Diagnostic Confidence Index (Robertson et al., 1999), while tic severity was determined with the Yale Global Tic Severity Scale (YGTSS) (Leckman et al., 1989). To further quantify tic symptoms, a standardized video recording was taken and was independently scored by two experienced physicians using the Modified Rush Videotape Rating Scale with scores ranging from 0 to 20 (Goetz et al., 1999). In cases where the reviewers’ scores differed, a consensus score was reached through a review of the relevant video segments. Overall tic frequency (tics per minute) was also determined.

For the GTS group, 15 patients had at least one psychiatric comorbidity (mood disorders (n = 8), ADHD (n = 5) or OCD (n = 4)), and 10 patients received pharmacological treatment, including: aripiprazole (n = 3), methylphenidate (n = 3), tiapride (n = 2) and the following medications each in one patient (n = 1): amitriptyline, prothipendyl, opipramol, atomoxetine. The average disease duration was 12.47 years (*SD* = 10.69). The mean Total Yale Tic Severity Score was 18.87 (*SD* = 9.72), and the Yale Global score was 33.98 (*SD* = 18.77). The Diagnostic Confidence Index yielded a mean score of 52.98 (*SD* = 19.56). The mean Rush Score was 10,95 (*SD* = 3.60) and mean motor tic frequency 37.37 tics per minute (*SD* = 25.03). As for the HC group, one participant was diagnosed with anorexia nervosa and another with depression. Only one participant was on medication (venlafaxine). The detailed clinical characteristics comparison are summarized in Table 1.

The study received approval from the local ethics committees, and all participants provided written informed consent prior to participation. For pediatric and adolescent participants, informed consent was obtained from their legal guardians. The research adhered to the ethical guidelines outlined in the Declaration of Helsinki.

### 2.2 Task

A well-established Stimulus (S)-Response (R) event file task (Colzato et al., 2006) was used. The task was displayed on a 25-inch monitor, with participants seated 60 cm away. Each trial began with a central fixation cross presented for 1000 to 1500 ms, followed by a cue in the form of an arrow (left or right) embedded in the center of a rectangular frame with three evenly spaced boxes. The cue remained visible for 1500 ms before the screen turned black for 1000 ms. Participants were instructed to remember the arrow’s direction and respond when the upcoming prime stimulus (S1) appeared in one of the outer boxes (top or bottom) as a horizontally or vertically oriented bar filled with either red or green. Participants had a maximum of 500 ms to respond as quickly and accurately as possible at S1 based on the direction of the cue (arrow), which was referred to as R1. Thus, the left index finger had to press a left key when the cue was a left-pointing arrow, while the right index finger had to press a right key when the cue was a right-pointing arrow. Importantly, S1 served as a stimulus when to react (R1) but did not instruct the type of movement, which was instructed by the cue. Then, the screen turned black for 2000 ms before the probe stimulus (S2) appeared, which shared the same feature dimensions (location, orientation, and color) as S1. The S2 stimulus disappeared as soon as participants responded (R2) based on the orientation of the target bar: a horizontal bar required pressing the left key with the left index finger, while a vertical bar required pressing the right key with the right index finger. The maximum response window was 2000 ms. Both reaction times and error rates were recorded for responses to both S1 (R1) and S2 (R2) stimuli.

The S-R task consisted of a total of 384 trials, which were equally divided into 6 blocks. These trials encompassed 16 conditions, defined by the response relation (either repeated or alternated) and the feature relation (with one, two, or all three features repeated or alternated) between S1 and S2. Focusing solely on the trials with full feature repetition or alternation, the key conditions included Response Repetition with Feature Alternation (RRFA), Response Repetition with Feature Repetition (RRFR), Response Alternation with Feature Alternation (RAFA), and Response Alternation with Feature Repetition (RAFR). According to BRAC framework, once R1 was completed, the features consisting of the R1 response (left or right index finger click) and the stimuli S1 (three distinct features) would be stored together in an event file, representing the process of binding. In the cases of RRFA, RRFR, and RAFA, since at least one feature from the previous event file was repeated, it would be extracted when S2 was presented, marking the process of retrieval. When the S2-R2 pairing did not align with the S1-R1 pairing (as in RRFA and RAFR), a new event file would be reconfigured, resulting in longer reaction times or increased error rates, a phenomenon referred to as partial repetition costs. In contrast, under the fully repeated condition (RRFR), a faster reaction time and fewer errors would be expected because no reconfiguration is required. In the RAFA condition, reaction times should also be faster and error rates lower compared to the RRFA and RAFR conditions given that no retrieval processes occur.

### 2.3 EEG recordings and pre-processing

EEG recordings were obtained using 60 Ag/AgCl electrodes arranged in an equidistant layout on EEG caps (EasyCap, Wörthsee, Germany), in conjunction with a BrainAmp DC amplifier (Brain Products, Gilching, Germany). The sampling rate for the recordings was set to 500 Hz. Electrode impedances were maintained below 5 kΩ throughout the experiment. The reference and ground electrodes were positioned at coordinates θ = 58, φ = 78 and θ = 90, φ = 90, respectively.

The raw recording data were then manually pre-processed using the Brain Vision Analyzer software (Brain Products, Gilching, Germany). Initially, the raw data were down-sampled to 256 Hz. Subsequently, an Infinite Impulse Response (IIR) filter with a passband of 0.5–40 Hz (order 8) was applied, along with a 50 Hz notch filter to remove power line interference. All channels were then visually inspected to identify and exclude any faulty channels before re-referencing, with the new reference calculated as the average of all functional channels, excluding the original reference. The raw data inspection was subsequently performed to manually remove synchronized artifacts across channels, such as muscle artifacts, as well as any periods corresponding to breaks between the experimental blocks. Following the first step of artifact removal, Independent Component Analysis (ICA) was applied using the Infomax algorithm to isolate and remove components associated with common artifacts, such as eye blinks, lateral eye movements, and cardiac activity. Another manual inspection of the raw data was conducted afterward to ensure the removal of any residual artifacts. For channels that were previously excluded, spherical spline interpolation (order 4) was applied to reconstruct the signals. The pre-processed data were then segmented into epochs with a length of 9000 ms, centered around the S2 stimulus (−7000 ms to 2000 ms). Automated artifact rejection was employed to remove epochs displaying large amplitude differences (greater than 200 μV within 200 ms), extreme values (exceeding ±200 μV within 200 ms), or low activity levels (below 0.5 μV per 100 ms). The remaining epochs (mean value: 51.07 ± 16.77) were exported to MATLAB (R2022b) using the FieldTrip toolbox (Oostenveld et al., 2010) for further analysis.

### 2.4 Time-frequency analysis

The preprocessed data were segmented into pre-trial (−7000 to 0 ms) and post-trial (0 to 2000 ms) periods relative to the S2 stimulus. Consistent with the behavioral analysis outlined in Section 2.2, the post-trial segments were further categorized based on the within-subject factors (Response and Feature relations) into the following conditions: RRFR, RRFA, RAFR, and RAFA. Time-frequency analyses were performed on both the pre-trial and post-trial periods using Morlet wavelets (width = 5). To assess potential binding effects, the average power differences between Feature Repetition (FR) and Feature Alternation (FA) were calculated separately under the conditions of Response Repetition (RR) and Response Alternation (RA) for both the GTS and HC groups. For a detailed spatial analysis, multiple t-tests were conducted at each electrode within three frequency bands – theta (4–7 Hz), alpha (8–12 Hz), and beta (13–30 Hz) – comparing FR and FA conditions in the 0 to 1000 ms time window for both groups. The results were corrected for multiple comparisons using the Benjamini-Hochberg-method (Benjamini and Hochberg, 1995) to control for the false discovery rate (FDR). Only results for conditions with statistically significant channels (p < 0.05) were plotted.

### 2.5 Beamforming analysis

Two beamforming techniques were sequentially applied to the pre-processed data in the sensor-level using the FieldTrip toolbox in line with previous studies (Adelhöfer and Beste, 2020, Rawish et al., 2024, Wendiggensen and Beste, 2023). In the first step, Dynamic Imaging of Coherent Sources (DICS) (Gross et al., 2001) beamforming was employed to project the sensor-level data onto the source-level in the frequency domain. For each subject, a standard Boundary Element Method (BEM) head model was aligned with the electrode layout and used to compute the leadfield at each point of the source grid (3D grid with a resolution of 0.5 cm). The frequency spectra for the theta, alpha, and beta bands were estimated using the Fast Fourier Transform (FFT) with a Hanning taper, yielding cross-spectral density (CSD) matrices. A common spatial filter, computed across all conditions using the CSD matrix and the leadfield to optimize source localization, was applied separately to pre-trial and post-trial data for each frequency band. For the pre-trial period, two time intervals of interest (TOIs) were defined: post-S1 (−2500 to −1500 ms) and pre-S2 (−1000 to 0 ms). The average power across HC and GTS participants was calculated for the three frequency bands (alpha, beta, and theta) within these TOIs. For the post-trial period, specifically post-S2 (0 to 1000 ms), the interaction between FR and FA was normalized separately for RR and RA across the same three frequency bands, using the following equation.

$$\mathrm{ratio}=\frac{\mathrm{powerFR}-powerFA}{\mathrm{powerFR}+powerFA}$$

To further identify coherent brain regions within each frequency band for both pre- and post-trial periods, the Density-Based Spatial Clustering of Applications with Noise (DBSCAN) algorithm (Ester et al., 1996) was applied to the average data across the HC and GTS groups, derived from the results of the DICS beamforming method. The top 1 % of the power distribution within voxels in structures with a label in the Automatic Anatomical Labeling (AAL) atlas (excluding the cerebellum) were selected for the clustering algorithm. The minimum number of voxels required to form a cluster was set at 2, with the lead field grid size set at 0.5 cm. The Epsilon (neighborhood search radius) was defined as 1.5 times the grid size to ensure the inclusion of all neighboring voxels at the edges. For the Pre-trial period, clusters were detected for both the Post-S1 and Pre-S2 segments across all three frequency bands. For the Post-trial period, the algorithm was applied only to conditions with significant channels in specific frequency bands, as determined by t-tests after frequency analysis at the sensor level in the last section.

In the subsequent stage, source-level data with time series information were reconstructed using the Linear Constraint Minimum Variance (LCMV) beamforming method. The source-model derived from the DBSCAN algorithm was imported from the previous stage. Spatial filters were then calculated for each dataset, constrained by TOIs (Post-S1, Pre-S2 and Post-S2) based on the covariance matrix from the time-locked analysis. The same time–frequency analysis using the wavelet method was then applied to the filtered average data within each cluster. For the post-S2 data, the interaction of the within-subject factors was computed as the difference between FR and FA under the conditions of RR and RA, indicating potential binding effects. Finally, Pearson correlation matrices between pairs of pre-trial clusters (Post-S1 and Pre-S2) and post-trial clusters were computed within the TOIs for both the GTS and HC groups. Each element of the matrix represented the correlation coefficient (r-values) between the given pair of clusters at each time point. The corresponding significance matrices (p-values) were corrected to q-values using the Benjamini-Hochberg method to account for potential false discovery rates (FDR) due to the large number of significance tests. Additionally, the significance threshold was set at 0.01.

### 2.6 Statistical analysis

**Fig. 1:**
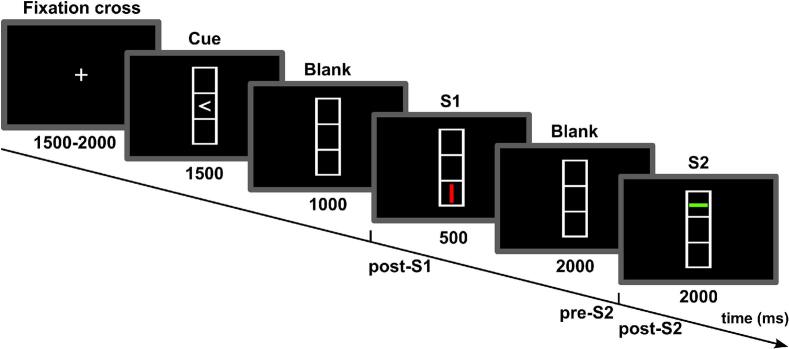
Schematic of a single trial in the Stimulus–Response (S–R) event-file task. Each trial began with a fixation cross (1500–2000 ms), followed by a cue (1500 ms) and a blank screen (1000 ms). The prime stimulus (S1) was then presented for a maximum of 500 ms or until a response (according to the direction of cue) was made. After a 2000 ms blank interval, the probe stimulus (S2) appeared, requiring a response within 2000 ms. EEG segments were time-locked to the onset of S2, with analysis windows defined as Post-S1 (1000 ms after S1 onset), Pre-S2 (1000 ms before S2 onset), and Post-S2 (1000 ms after S2 onset).

**Fig. 2:**
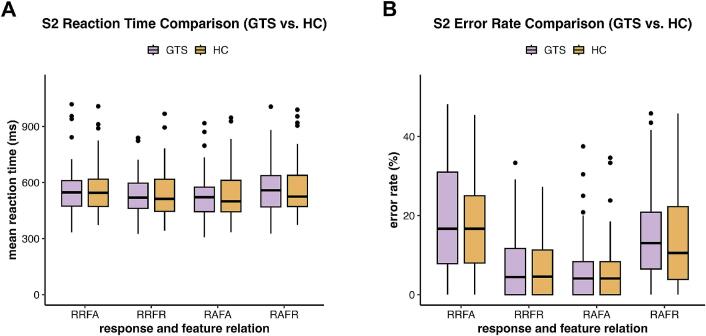
Mean reaction time (panel A) and error rate (panel B) for the GTS and HC groups across the four response–feature relation conditions: Response Repetition with Feature Alternation (RRFA), Response Repetition with Feature Repetition (RRFR), Response Alternation with Feature Alternation (RAFA), and Response Alternation with Feature Repetition (RAFR). (A) Mean reaction time for the GTS group (purple) and the HC group (orange). (B) Error rate for the GTS group (purple) and the HC group (orange). (For interpretation of the references to color in this figure legend, the reader is referred to the web version of this article.)

**Fig. 3:**
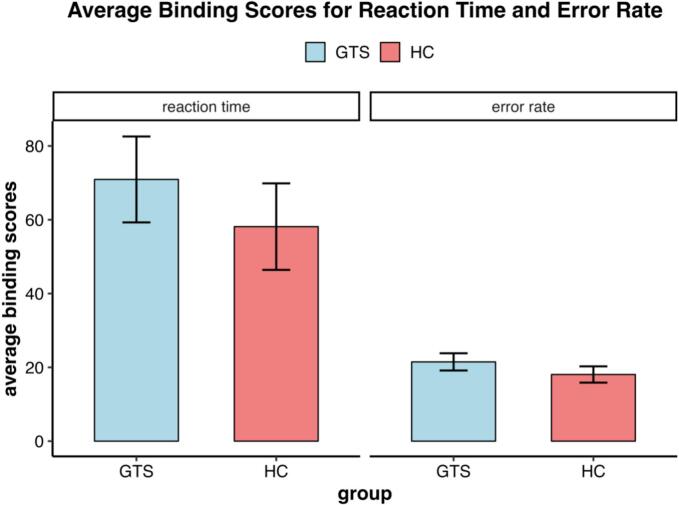
Average binding scores for reaction times and error rates in the GTS and HC groups.

The behavioral data analysis was conducted in RStudio (version 2023.12). Only trials involving fully repeated or fully changed features (S2 compared with S1) were included in the analysis. During data preprocessing, trials with incorrect or missed responses to S1 were excluded. Additionally, for each participant, trials where S2 response times were lower than 200 ms or higher than the upper boundary defined by Tukey’s fences (Q3 + 1.5 * IQR) were excluded. Mean reaction times and error rates (the percentage of incorrect trials) for S2 were computed and analyzed using repeated measures analysis of variance (ANOVA) with Type III sums of squares. The within-subject factors (RRFA, RRFR, RAFA and RAFR) included the response relation and feature relation between S1 and S2, which were used to evaluate potential binding effects. Group categorization was included as a between-subjects factor to compare differences between GTS and HC. To further evaluate the binding effect between the GTS and HC groups, binding scores were calculated for both reaction times and error rates using the formula: ((RRFA − RRFR) + (RAFR − RAFA)), which represents the benefit of feature repetition in both the RR and RA conditions. Higher positive binding scores indicated stronger binding effects. Initially, one-sample t-tests were conducted to assess the presence of binding effects within each group (GTS and HC). Subsequently, the binding scores between the GTS and HC groups were compared using Welch's t-tests and Bayesian analysis (Fig. 1, Fig. 2, Fig. 3).

## 3 Results

### 3.1 Behavioral data

A three-way ANOVA on S2 mean reaction times revealed a significant interaction between response relation and feature relation, *F*(1,114) = 60.37, *p* < 0.01, *η^2^g* = 0.015, indicating a significant binding effect. However, there was no significant main effect of group, *F*(1,114) = 0.09, *p* = 0.76, *η^2^g* < 0.01, nor a significant three-way interaction between response relation, feature relation, and group, *F*(1,114) = 0.59, *p* = 0.44, *η^2^g* < 0.01. No significant main effects were observed for response relation, *F*(1,114) < 0.01, *p* = 0.99, *η^2^g* < 0.01, or feature relation, *F*(1, 114) = 2.09, *p* = 0.15, *η^2^g* < 0.01. Within-group post-hoc t-tests revealed significant binding effects when comparing average binding scores against zero in both the HC group (*M* = 58.14, *SD* = 92.31, *t*(61) = 4.96, *p* < 0.01, *dz* = 0.63) and the GTS group (*M* = 70.93, *SD* = 85.56, *t*(53) = 6.09, *p* < 0.01, *dz* = 0.83). However, the between-group comparison revealed no significant difference between the GTS and HC groups, *t*(113.54) = 0.77, *p* = 0.44, *d* = 0.14, BF_10_ = 0.26 ± 0.03 %.

In the analysis of error rates, the three-way ANOVA revealed a significant interaction between response relation and feature relation, *F*(1,114) = 151.42, *p* < 0.01, *η^2^g* = 0.18. There was no significant main effect of group, *F*(1,114) = 0.86, *p* = 0.36, *η^2^g* < 0.01, nor a significant three-way interaction, *F*(1,114) = 1.13, *p* = 0.29, *η^2^g* < 0.01. Significant main effects were found for both response relation, *F*(1,114) = 11.09, *p* < 0.01, *η^2^g* = 0.02, and feature relation, *F*(1,114) = 7.10, *p* < 0.01, *η^2^g* = 0.01. Significant binding effects were observed in both the HC group (*M* = 18.06, *SD* = 17.36), *t*(61) = 8.19, *p* < 0.01, *dz* = 1.04, and the GTS group (*M* = 21.48, *SD* = 17.16), *t*(61) = 9.20, *p* < 0.01, *dz* = 1.25, as demonstrated by the within-group post-hoc t-tests. However, the between-group comparison showed no significant difference between the GTS and HC groups, *t*(112.16) = 1.07, *p* = 0.29, *d* = 0.20, with BF_10_ = 0.33 ± 0.03.

To account for potential confounding effects of age, comorbidity (GTS group), medication (GTS group) and IQ, repeated-measures analysis of covariance (ANCOVAs) were conducted using each factor separately as a covariate. The analysis revealed no significant interaction among age, response relation, and feature relation on reaction time (*F*(1,113) = 0.0ß, *η^2^_g_* < 0.01, *p* = 0.78). However, a significant interaction was found between response relation and feature relation (*F*(1,113) = 14.3, *η^2^_g_* < 0.01, *p* < 0.01). Regarding error rate, no significant interaction was observed among age, response relation, and feature relation (*F*(1,113) = 1.95, *η^2^_g_* < 0.01, *p* = 0.17, whereas the interaction between response relation and feature relation remained significant (*F*(1,113) = 47.34, *η^2^_g_* = 0.07, *p* < 0.01). These results suggest that age differences did not significantly influence the binding effect. Likewise, comorbidity, medication and IQ showed no significant influence (i.e., no significant three-way interaction among covariate, response relation, and feature relation) on either reaction time or error rate: Comorbidity (reaction time: *F*(1,51) = 0.29, *η^2^_g_* < 0.01, *p* = 0.59; error rate: *F*(1,51) = 3.99, *η^2^_g_* = 0.01, *p* = 0.51), Medication (reaction time: *F*(1,52) = 0.03, *η^2^_g_* < 0.01, *p* = 0.87; error rate: *F*(1,52) = 0.44, *η^2^_g_* < 0.01, *p* = 0.51), IQ (reaction time: *F*(1,113) = 0, *η^2^_g_* < 0.01, *p* = 0.99; error rate: *F*(1,49) = 1.59, *η^2^_g_* < 0.01, *p* = 0.21).

### 3.2 Neurophysiological data: post-trial period (Post-S2)

**Fig. 4:**
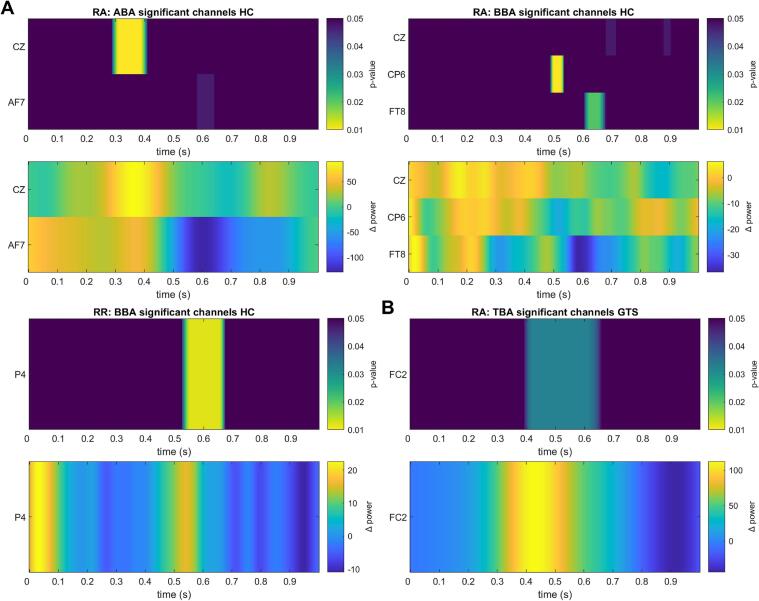
Significant channels in the sensor level. RA: response alternation; RR: response repetition; TBA: theta-band activity; ABA: alpha-band activity; BBA: beta-band activity. (A) HC group under RA or RR conditions; (B) GTS under RA condition.

**Fig. 5:**
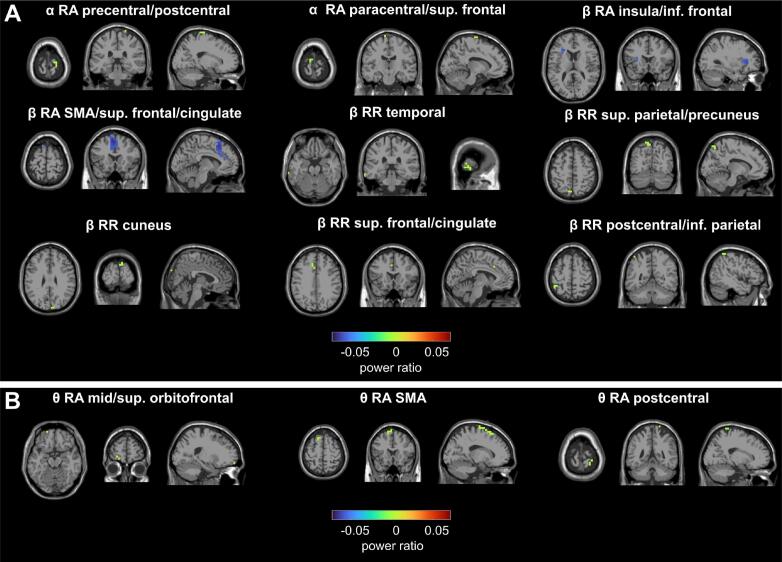
Significant clusters of power ratio changes across frequency bands (theta, alpha, and beta) during the post-trial period (0–1000 ms) under the condition of response alternation (RA) or response repetition (RR). (A) In healthy controls (HC), significant clusters were identified in the alpha-band (precentral/postcentral gyrus, paracentral lobule, and superior frontal gyrus) and the beta-band (supplementary motor area (SMA), superior frontal gyrus, cingulate cortex, insula, inferior frontal gyrus, temporal cortex, superior parietal lobule/precuneus, postcentral gyrus/inferior parietal lobule, cuneus, and superior frontal/cingulate regions). (B) In GTS, significant clusters emerged in the theta-band, involving the middle/superior orbitofrontal cortex, SMA, and postcentral gyrus. The color bar indicates power ratio values, with warm colours representing increases and cool colours representing decreases.

In the HC group, a significant positive power difference (*P*_FR_ > *P*_FA_, *p* < 0.01) between Feature Repetition (FR) and Feature Alternation (FA) trials was found at channel P4 from 550 ms to 650 ms under the Response Repetition (RR) condition in the beta-band after the onset of S2, indicating increased activity in the right parietal region (Fig. 4A). The DBSCAN algorithm detected few significant beta-band clusters at the source level, primarily distributed in the temporal, parietal and frontal regions of the left hemisphere. These clusters included activation in the middle and inferior temporal gyrus, superior parietal lobule, precuneus, cuneus, superior frontal gyrus, cingulate cortex, postcentral gyrus, as well as inferior parietal regions (Fig. 5A), which reflected the active cortical region involved in the dynamic management of the event file (retrieval or reconfiguration) triggered by the binding effect following the appearance of S2.

Under the Response Alternation (RA) condition, significant negative power differences (*P*_FR_ < *P*_FA_, *p* < 0.03) were observed at channels CP6 and FT8, from 500 ms to 550 ms and 600 ms to 700 ms followed the appearance of S2, respectively, within the beta-band, suggesting reduced neural activity during the FR condition compared to the FA condition (Fig. 4A). Significant beta-band clusters were revealed using DBSCAN algorithm under the RA condition involving the insula, and extensive regions of the frontal cortex, including the inferior frontal gyrus, supplementary motor areas, and the anterior and middle cingulate cortices at the source level (Fig. 4A). In the alpha-band, channel Cz demonstrated a significant positive power differences between the FR and FA from 300 ms to 400 ms. Furthermore, two significant alpha-band clusters were detected at the source level: one in the precentral and postcentral gyri in the right hemisphere, indicating somatosensory activity, and another in the paracentral lobule and superior frontal gyrus in the left hemisphere suggesting motor and higher-order cognitive engagement (Fig. 5A).

In the GTS group, however, a significant effect was found only in the theta-band at the sensor level, based on FDR-corrected t-tests comparing the results of time–frequency analysis between FR and FA. Specifically, a significant positive difference (*P*_FR_ > *P*_FA_, *p* < 0.04) was observed at channel FC2 under RA condition, occurring between around 400 ms and 650 ms after the onset of S2 (Fig. 4B). Correspondingly, at the source level, the DBSCAN algorithm identified three significant theta-band clusters in the frontal and parietal cortices of both hemispheres, including the middle and superior frontal gyri, supplementary motor areas, and parietal lobules under the RA condition (Fig. 5B). In comparison to the HC group, no significant channels were detected in the alpha or beta frequency bands at the sensor level.

### 3.3 Neurophysiological data: pre-trial period (post-S1 and Pre-S2)

**Fig. 6:**
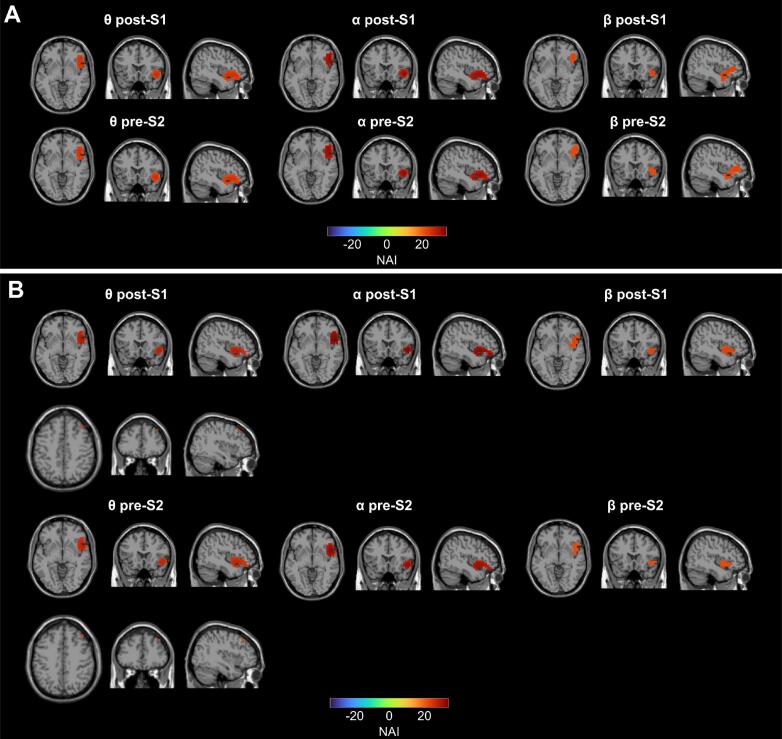
Significant clusters across different frequency bands (theta, alpha and beta) during the pre-trial periods (post-S1: −2500 to −1500 ms; pre-S2: −1000 to 0 ms). NAI: Neural Activity Index. (A) Clusters detected in HC: theta- and alpha- band clusters in the insula, inferior frontal and superior temporal regions; beta-band clusters in the insula, inferior/middle frontal and superior/middle temporal cortices. No differences were observed between the post-S1 and pre-S2 periods, apart from the number of voxels. (B) Clusters detected in GTS: similar to HC, with an additional theta-band cluster in the middle frontal gyrus.

Consistent with our previous publication (Rawish et al., 2024), a direct comparison analysis between and FR and FA trials at the sensor level was not feasible for the pre-trial timeframe, as response–feature relations had not yet been established at this stage. Following source reconstruction, the DBSCAN algorithm identified significant power clusters (top 1 % of the strongest voxels) across all three frequency bands during the post-S1 and pre-S2 period in both groups. In the theta and alpha frequency bands, the identified clusters showed only minor differences in voxel count and were predominantly located in the right insula, inferior frontal gyrus, and superior temporal cortex in both the GTS and HC groups during the post-S1 and pre-S2 periods (Fig. 6). Notably, the GTS group exhibited an additional theta-band cluster in the right middle frontal gyrus (Fig. 6B, second rows of θ post-S1 and θ pre-S2), which was not present in the HC group. In the beta frequency band, significant clusters were observed in the insula, inferior/middle frontal gyrus, and superior/middle temporal cortices in both groups across both time intervals.

### 3.4 Neurophysiological data: correlation analysis

Correlation analyses were performed on source-reconstructed time series data within the clusters identified in the previous sections (Fig. 5, Fig. 6), focusing on the interaction between Pre-trial (encompassing Post-S1 and Pre-S2; corresponding to event file integration and maintenance as conceptualized within the BRAC framework) and Post-trial (Post-S2; corresponding to event file retrieval or reconfiguration) periods. Analyses were conducted separately for the HC and GTS groups.

In the HC group, significant correlations were primarily observed between post-trial beta-band clusters and pre-trial clusters across the theta, alpha, and beta frequency bands. In contrast, in the GTS group, significant correlations were mainly found between post-trial theta-band clusters and pre-trial clusters. The complete set of significant correlation patterns is provided in the Supplementary material. Of note, the post-trial cluster in the supplementary motor area (SMA) exhibited significant correlations with pre-trial clusters in both groups.

**Fig. 7:**
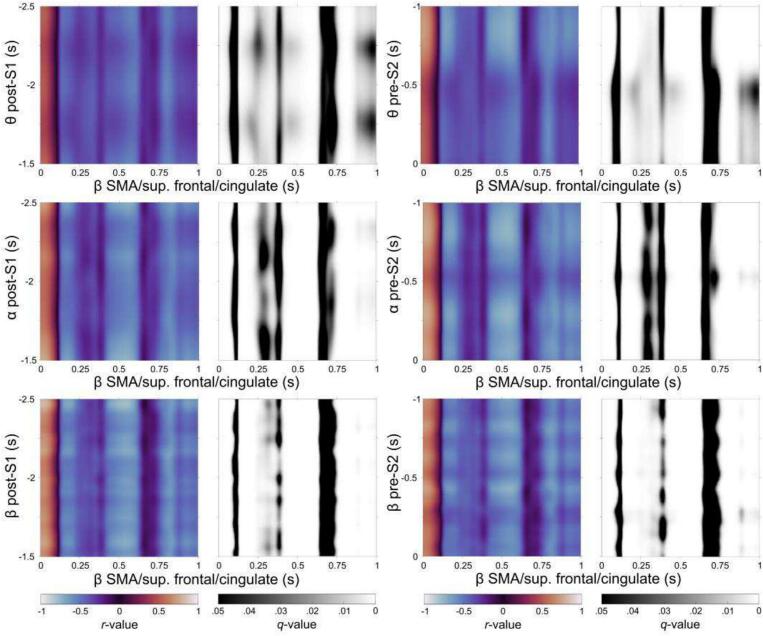
Significant correlations between the post-trial beta-band cluster in SMA/superior frontal/cingulate and pre-trial clusters across three frequency bands (theta, alpha and beta) in HC.

In HC (Fig. 7), beta oscillatory activity in the SMA following the S2 response showed significant correlations with theta-band clusters throughout the entire Post-S1 (*r_max_* = 0.56; *r_min_* = −0.57; within area, *q* < 0.05) and Pre-S2 (*r_max_* = 0.73; *r_min_* = −0.73; within area, *q* < 0.05) periods. Notably, the correlations were positive up to approximately 100 ms following S2 onset, after which they transitioned to negative. This pattern was also observed in alpha-band (Post-S1: *r_max_* = 0.78; *r_min_* = −0.75; within area, *q* < 0.05; Pre-S2: *r_max_* = 0.78; *r_min_* = −0.75; within area, *q* < 0.05) and beta-band (Post-S1: *r_max_* = 0.82; *r_min_* = −0.80; within area, *q* < 0.05; Pre-S2: *r_max_* = 0.75; *r_min_* = −0.74; within area, *q* < 0.05) activities across the entire pre-trial period.

**Fig. 8:**
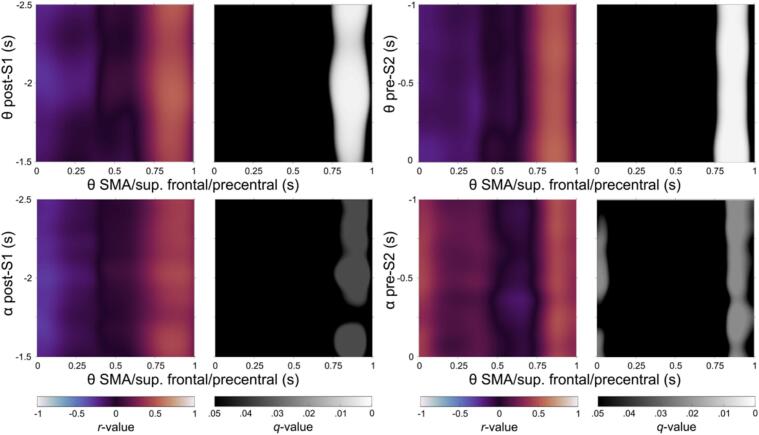
Significant correlations between the post-trial beta-band cluster in SMA/superior frontal/precentral and pre-trial theta- and alpha-band clusters in GTS.

In GTS (Fig. 8), theta oscillations during the post-S1 period showed significant correlations with a theta-band cluster in the post-trial period within the SMA, approximately from 750 to 950 ms (*r_max_* = 0.66; *r_min_* = −0.35; within area, *q* < 0.05). A similar pattern was observed between pre-S2 and post-S2 theta-band clusters in the same cortical region (*r_max_* = 0.64; *r_min_* = −0.40; within area, *q* < 0.05). Additionally, alpha oscillations during the post-S1 period were significantly correlated with theta activity in the SMA during the post-S2 time window from 750 to 950 ms (*r_max_* = 0.62; *r_min_* = −0.36; within area, *q* < 0.05). Comparable correlations were also found during the pre-S2 period (*r_max_* = 0.62; *r_min_* = −0.43; within area, *q* < 0.05). However, no significant correlations were observed between beta-band clusters in the pre-trial and post-trial periods.

The results of the analyses of other brain regions are shown in Supplemental Figs. 1 and 2.

## 4 Discussion

The current study focused on the effect of the immediate past (pre-trial period) on the management of integrated sensorimotor representations (event files, post-trial period). Changes in the processing of event files have been considered to be central for a better understanding of GTS on a mechanistic level (Adelhöfer et al., 2021, Beste et al., 2021, Beste and Münchau, 2018, Kleimaker et al., 2020b, Münchau et al., 2024, Petruo et al., 2019). However, so far, the understanding of the dynamics of sensorimotor integration in GTS has been incomplete. This is mainly the case because the build-up of sensorimotor representations critically depends upon the immediate past of sensorimotor processes (Beste et al., 2023, Frings et al., 2020, Frings et al., 2024). A better understanding of sensorimotor dynamics in GTS must therefore consider this dynamic.

Using a standard experimental approach to study event file processing (Colzato et al., 2006, Hommel, 2004) and the effects of the immediate past on sensorimotor processes, we examined theta, alpha and beta band activity in the present study in patients with GTS and HC in a similar way as recently in a study of HC (Rawish et al., 2024).

On a behavioral level, there were standard binding effects in each of the examined groups, but no significant group difference in the strength of binding. At first sight this seems to be at odds to previous findings suggesting altered behavioral binding effects in patients with GTS (Kleimaker et al., 2020b, Petruo et al., 2019). However, it has to be considered that the current study comprised a much larger age range including not only adult patients with (persisting) GTS, but also adolescents with GTS. The latter have a different pattern of modulation in event file binding compared to adults with GTS (Beste et al., 2021), which explains the lack of behavioral effects in this large cohort of adolescents and adults.

The analysis of the neurophysiological data after the S2 stimulus presentation shows a well-known pattern of activity replicating previous studies in the field. It is shown that theta, alpha and beta band activity is involved during the retrieval of an already established event file. Likewise, also the functional neuroanatomical regions identified corroborate previous findings (for review see (Beste et al., 2023). In particular, regions in the superior frontal cortex encompassing the SMA, the anterior cingulate cortex, superior and inferior parietal cortices as well as areas constituting the ventral visual stream were evident. However, of primary importance for the goal of the current work is what happens in the S1-S2 interval on the neurophysiological level and how this relates to neurophysiological processes after the S2 stimulus presentation.

Replicating previous findings in healthy individuals (Rawish et al., 2024), we found that once an event file was established after the presentation of the S1 stimulus and before this event file was processed again (i.e., at the S2 stimulus), theta, alpha and beta band activity was evident in the insula cortex, inferior/middle frontal and superior/middle temporal areas of the right hemisphere in HC (Fig. 6A). Similar activity patterns in the same brain regions were also present in patients with GTS (Fig. 6B), who had an additional theta-band cluster in the right middle frontal gyrus. The inferior frontal cortex and the anterior part of the superior temporal cortex play a role in the processing of sensorimotor features for purposeful actions (Borra et al., 2017). Recent evidence suggests a more general role of these regions during sensorimotor integration processes (Mayer et al., 2025). More precisely, it was shown that inferior frontal and anterior temporal cortices plus insular cortices build a close network of direct information transfer to maintain a recently created event file. This is reasonable because the insular cortex plays a prominent role in sensorimotor integration (Cauda et al., 2012, Droutman et al., 2015, Gogolla, 2017), and the anterior temporal cortex possibly constitutes a hub region (Binney et al., 2016, Hoffman et al., 2015) to distill coherent (action) concepts from multimodal inputs (Rice et al., 2015), possibly because the anterior part of the temporal cortex is part of the ventral stream pathway involved in the processing of the identity of visual features (Chao and Martin, 1999, Goodale et al., 2005, Goodale and Milner, 1992). While this has been suggested on the basis of the analysis of theta band activity only (Mayer et al., 2025), it is reasonable that the alpha and beta activity reveal similar activity patterns because all three frequency bands likely interact during the dynamic management of event files (Beste et al., 2023).

The fact that both patients with GTS and HC had the same activity pattern in these regions is important, particularly against the background that changes in functional neuroanatomical activity and connectivity patterns are frequently observed in GTS in multiple neural systems and especially those associated with action control. Notably, however, such changes were mostly observed in fronto-parietal networks. From that perspective, it appears that “canonical regions” important for sensorimotor integration in insular cortex, anterior temporal lobe, and the inferior frontal cortex (Mayer et al., 2025) remain relatively intact in GTS. Crucially, however, this only refers to the local activity pattern, but not to how the neural activity patterns (i.e. activity in the observed brain regions) depend on each other across time. This information was revealed by the correlation analyses (Fig. 7, Fig. 8).

There was a correlation of theta band activity post-S1 and pre-S2 with theta band activity post-S2 in patients with GTS during the first 750 ms after the presentation of the S2 stimulus. Several lines of evidence suggest that theta band activity during the first 750 ms is important for the efficient retrieval of event files (Jamous et al., 2024, Rawish et al., 2024, Wendiggensen et al., 2023). This finding is of pathophysiological importance given that theta band activity has been suggested to be key to the understanding of GTS pathophysiology (Alam et al., 2015, Cagle et al., 2020, Giorni et al., 2017, Jimenez-Shahed et al., 2016, Maling et al., 2012, Marceglia et al., 2010, Molina et al., 2018, Münchau et al., 2021, Neumann et al., 2018, Priori et al., 2013, Shute et al., 2016). The observed pattern in patients with GTS thus suggests a qualitative change in the neurophysiological architecture underlying binding and retrieval mechanisms during sensorimotor integration.

Considering the correlations between the S1-S2 interval (post-S1 and pre-S2) with post-S2 activity patterns in patients with GTS and healthy controls, especially the activity pattern post-S2 in the SMA/prefrontal cortex is of conceptual relevance. The reasons are that (i) this region is the only one consistently evident in the post-S2 period in GTS and healthy individuals, (ii) this region has previously been implicated in event files processing across tasks and methods addressing neural activity patterns underlying event file processing (Beste et al., 2023, Elsner et al., 2002), (iii) this region is of known pathophysiological relevance in GTS (Dyke et al., 2022, Finis et al., 2013, Tübing et al., 2018). In the GTS group, and the beta band, no significant correlations were detected within the first 750 ms following the onset of the S2 stimulus. In contrast, in HC substantial correlations between post-S1/pre-S2 and post-S2 activities were evident in the beta frequency band. Thus, neurophysiological differences in GTS compared to HC, evident during both the event-file binding and retrieval stages, are not confined to the theta-band.While the role of beta-band activity in the pathophysiology of GTS is unclear (but see: Morand-Beaulieu et al., 2023), it clearly plays an important role in the dynamic management of event files (Beste et al., 2023, Prochnow et al., 2022, Wendiggensen et al., 2023) both in binding and retrieval stages. The direction of correlations obtained shows that high beta-band activity after an event file has been bound/established incurs low activity in these frequency bands during the retrieval of these event files. This pattern might hint at differences in the termination of event-files (Frings et al., 2023). Especially beta band activity has been linked to the maintenance of S1 event files after the response to S1 (Pastötter et al., 2021). The basic idea is that only event files that are encapsulated are maintained and available for retrieval. In other words, only encapsulated event files underlie dynamic management processes and hence can be controlled/ re-configurated during retrieval. What we see in GTS patients might instead be the rest activation of not terminated event files that at the behavioral level prime feature and response repetitions (and mimic binding effects). This might be interpreted as one mechanism that consolidates tic behavior as event files containing tic behavior are not as controllable in GTS as other simple motor patterns are controllable in healthy controls.

The current findings suggest that the interplay of theta, alpha and beta band activity should be studied further to better understand the pathophysiology of GTS, especially as regards the novel concept of GTS as a disorder of perception–action integration.

In summary, we provided insights into the neural mechanisms underlying altered sensorimotor integration as a novel conceptual framing of GTS. While GTS patients and HCs share similar activity patterns in regions critical for sensorimotor integration, such as the insular cortex and anterior temporal lobe, GTS patients reveal a temporal disconnect of activity between different processing stages of sensorimotor integration. Sensorimotor integration is less reliant on the immediate past in GTS compared to HCs. This was particularly true for beta-band activity. However, distinct correlation patterns were also observed in the theta- and alpha-band activities, suggesting widespread temporal disconnection of information processing during sensorimotor integration in GTS.

## 5 Code availability statement

All codes can be obtained from the corresponding author upon reasonable request.

## Funding sources

This work was supported by a Grant from the 10.13039/501100001659Deutsche Forschungsgemeinschaft FOR 2698 and by 10.13039/501100002347Federal Ministry of Education and Research (Bundesministerium für Bildung und Forschung, BMBF) as part of the German Center for Child and Adolescent Health (DZKJ) under the funding code 01GL2405B.

## CRediT authorship contribution statement

**Yifan Hao:** Writing – review & editing, Writing – original draft, Visualization, Investigation, Formal analysis, Data curation. **Paul Wendiggensen:** Writing – review & editing, Writing – original draft, Visualization, Investigation, Formal analysis, Data curation, Conceptualization. **Annet Bluschke:** Writing – review & editing, Funding acquisition, Conceptualization. **Tina Rawish:** Writing – review & editing, Investigation. **Julia Friedrich:** Writing – review & editing, Investigation. **Eszter Tóth-Fáber:** Writing – review & editing, Resources. **Zsanett Tárnok:** Writing – review & editing, Resources. **Veit Roessner:** Writing – review & editing, Funding acquisition. **Christian Frings:** Writing – review & editing, Funding acquisition, Conceptualization. **Anne Weissbach:** Writing – review & editing, Funding acquisition. **Tobias Bäumer:** Writing – review & editing, Resources, Project administration. **Alexander Münchau:** Writing – review & editing, Writing – original draft, Validation, Resources, Project administration, Funding acquisition, Conceptualization. **Christian Beste:** Writing – review & editing, Writing – original draft, Supervision, Resources, Project administration, Methodology, Funding acquisition, Conceptualization.

## Declaration of competing interest

The authors declare that they have no known competing financial interests or personal relationships that could have appeared to influence the work reported in this paper.

## Data Availability

All data can be obtained from the corresponding author upon reasonable request.
