# Supplementary material for: Evidence for temporal disintegration of information processing during sensorimotor integration in GTS
Source: Neuroimage Clin. 2025 Jul 13;48:103844. doi: 10.1016/j.nicl.2025.103844 (PMC12296559; doi:10.1016/j.nicl.2025.103844)
Supplement: Supplementary Data 1 [file mmc1.docx]

**Supplemental Material**

**Evidence for temporal disintegration of information processing during sensorimotor integration in GTS**

Yifan Hao, Paul Wendiggensen, Annet Bluschke, Tina Rawish, Julia Friedrich, Eszter Tóth-Fáber, Zsanett Tárnok, Veit Roessner, Christian Frings, Anne Weissbach, Tobias Bäumer, Alexander Münchau, Christian Beste

*Supplementary Figure 1*


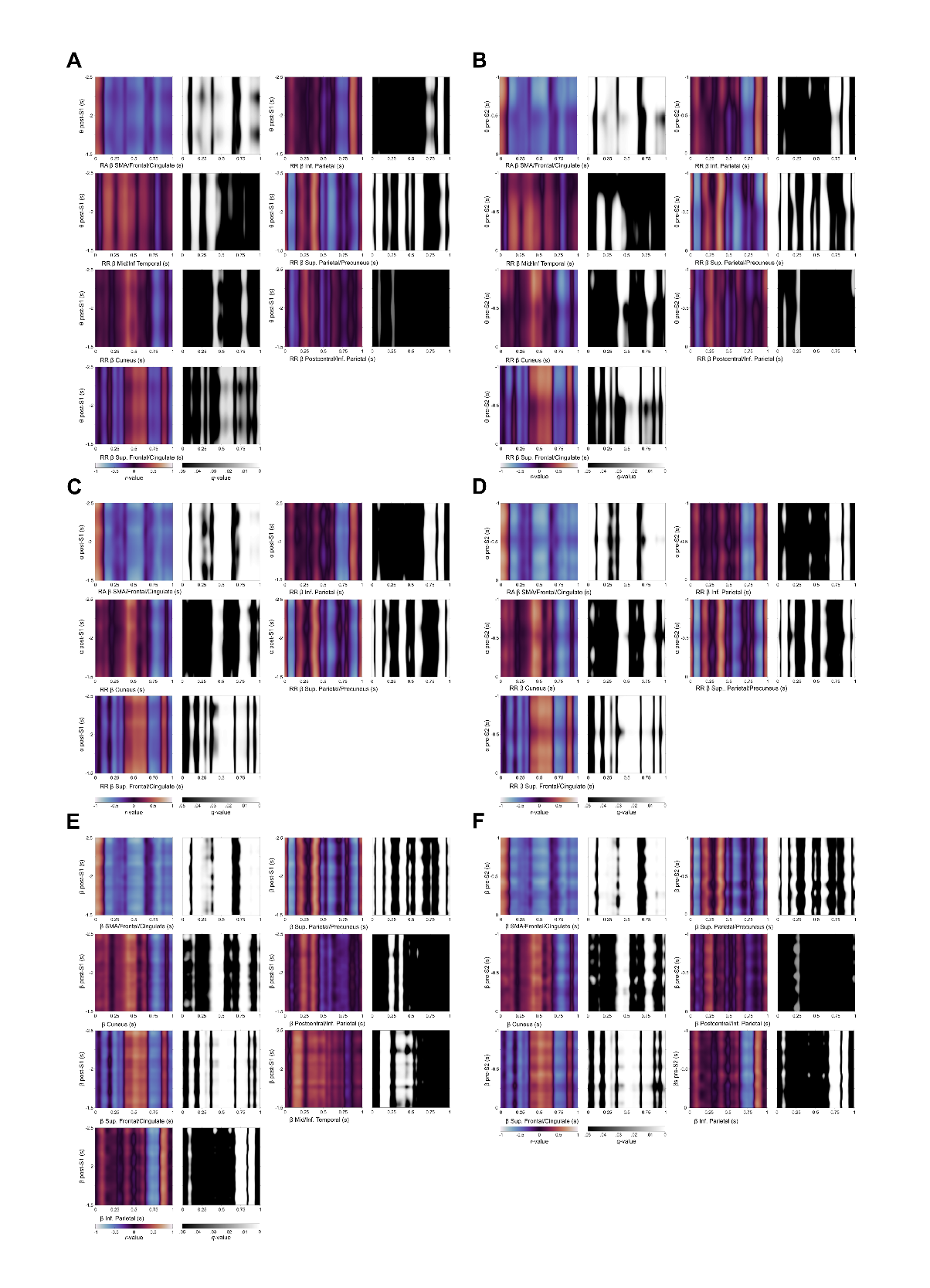


Significant correlation between the clusters across different time points for HC. (A) Post-S1 alpha band clusters and Post-S2 beta band clusters; (B) Pre-S2 alpha band clusters and Post-S2 beta band clusters; (C) Post-S1 beta band clusters and Post-S2 beta band clusters; (D) Pre-S2 beta band clusters and Post-S2 beta band clusters; (E) Post-S1 theta band clusters and Post-S2 beta band clusters; (F) Pre-S2 theta band clusters and Post-S2 beta band clusters.

*Supplementary Figure 2*


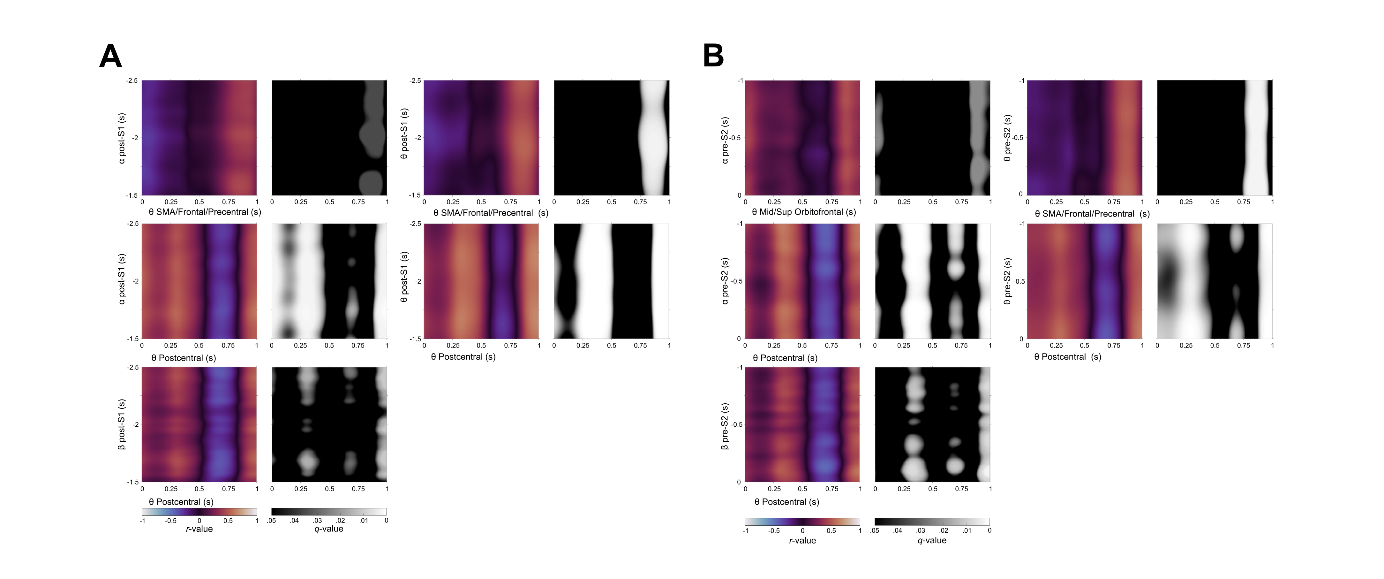


Significant correlation between the clusters across different time points for GTS. (A) Post-S1 clusters and post-S2 clusters in different frequency bands; (B) Pre-S2 clusters and post-S2 clusters in different frequency bands.
